# EP07 Eosinophilic granulomatosis with polyangiitis: diagnostic and therapeutic challenges during COVID-19 pandemic

**DOI:** 10.1093/rap/rkaa052.006

**Published:** 2020-11-03

**Authors:** Yvonne Tan, Ammar Mohamedalhadi, Fiona Wood

**Affiliations:** Furness General Hospital, Barrow, United Kingdom

## Abstract

**Case report - Introduction:**

COVID-19 pandemic affected medical practise significantly and caused difficulties in accessing necessary investigations at the appropriate time. As of March 2020, NHS England issued measures to redirect staffs and resources in preparation for the rising cases of coronavirus. As a result of this, non-urgent tests/treatments were put on hold. We present a new case of EGPA admitted to our district general hospital during the COVID-19 pandemic to highlight the challenges faced. The diagnosis was reached based on clinical judgment in the absence of some confirmatory tests as well as the decision of starting immunosuppressant treatment during the pandemic.

**Case report - Case description:**

A 41-years-old lady with a background of well-controlled asthma, presented with five days history of paraesthesia and swelling in both legs. She also reported mild pleuritic chest pain, which radiated to her left arm. Physical examination revealed left foot drop. She had reduced sensation on the L5-S1 dermatomal distribution with absent ankle reflex and reduced knee reflex of her left leg. Her left calf was swollen and tender. The rest of her examination was unremarkable.

Baseline blood revealed raised WCC of 19.3 with significant eosinophilia (10). CRP and ESR were 135 mg/L and 48mm/hr, respectively. Electrocardiogram showed new T-wave inversion in the anterolateral leads with significantly raised troponin levels. There was ground glass appearance in both lungs, keeping with suspected COVID-19 and no evidence of pulmonary embolus was found on CTPA. MRI spine confirmed no evidence of cauda equina compression. Deep vein thrombosis was also excluded with US doppler.

She was treated as myocarditis and pneumonia secondary to probable COVID-19 infection. Echocardiogram revealed severe LVSD (EF < 35%) with no LV hypertrophy. Three days later, she became acutely breathless and required high flow oxygen. New bilateral basal crackles were found on auscultation. Her antibiotic regimes were escalated to intravenous infusion.

A revised CT report suggested the findings may correlate with eosinophilic pneumonia or EGPA. MRI of lower legs proved muscular oedema in bilaterally, which was suggestive of myositis with fasciitis. There was no significant change on the thigh musculature. CK level was slightly elevated (403 IU/L). Urinalysis was positive for blood (3+). Given the strong clinical suspicion of EPGA, a decision to start high dose steroid therapy was made, despite the pending immunology results. After the third dose of the methylprednisolone, pulsed cyclophosphamide was started along with high dose oral prednisolone. The patient was discharged home following significant clinical improvement.

**Case report - Discussion:**

This patient has fulfilled 4 out of 6 criteria of ACR 1990 classification for EGPA, which are eosinophilia, bronchial asthma, mononeuritis multiplex and pulmonary infiltrates on radiological images. However, in the context of current pandemic, these changes on chest CT findings could also be suggestive of COVID-19 pneumonitis.

At present, there is no reliable test for COVID-19. Even though RT-PCR testing has been the gold standard for diagnosing suspected cases, the clinical sensitivity and specificity of these tests are variable. A negative test may not rule out infection. In our case, the patient was tested twice at separate times to rule out the possibility of COVID-19 infection.

During the pandemic, there is extremely limited access to some confirmatory tests. We were not able to perform nerve conduction studies on our patient as the service was suspended, instead, we sought neurologist’s review to confirm the mononeuritis multiplex. We also sought advice from haematologist to rule out the possibility of hyper-eosinophilic syndrome as bone marrow biopsy was unavailable. The screen for atypical pneumonia, aspergillosis, viruses, and tuberculosis were negative. By excluding the alternative diagnoses related to eosinophilia, we concluded that this was likely to be a case of first presentation EGPA.

Our next obstacle was introducing remission–induction regimens during COVID-19 pandemic. BSR does not recommend starting new treatment due to the increased risk of infection. We had to weigh out the benefits and risks of initiating immunosuppression. Our patient was made aware of the potential risks involved which include severe infection with COVID-19. She was also shifted to a side room with strict infection control precautions and PCP prophylaxis prescribed before starting pulsed methylprednisolone and cyclophosphamide. Fortunately, her neurological symptoms resolved after three days of steroid therapy. Eosinophils count dropped within 1 day to zero, after the first dose of IV methylprednisolone.

**Case report - Key learning points:**

Despite the rising cases of COVID-19 infection, it is essential to keep an open mind and consider alternative diagnosis if a patient did not respond to conventional treatment. As EGPA and COVID-19 pneumonia share similar clinical and radiological presentation, clinical judgement is essential when making the diagnosis as the treatments for both conditions are vastly different. When EGPA is suspected, a multidisciplinary team should be involved in the evaluation of different organ involvements as well as ruling out other causes of eosinophilia. The role of specialists’ inputs is extremely important in reaching the diagnosis, especially with limited access to the usual confirmatory tests due to reduced services during the pandemic.

In addition, when there is an increased risk of infection such as during the COVID-19 pandemic, it is essential to weigh up the benefits and risks of commencing immunosuppressant treatment carefully. Patients need to be involved in the decision-making process as well as take precautions to minimise the risk of infection. The decision to start remission induction regimes should not be delayed if there is a presence of life or organ threatening disease manifestations in EGPA patients. Our patient has had a life-threatening disease because of multi-organ involvements (cardiac, pulmonary, and neurological systems).

